# Case Report: Genomic Characteristics of the First Known Case of SARS-CoV-2 Imported From Spain to Sichuan, China

**DOI:** 10.3389/fmed.2021.783646

**Published:** 2021-11-30

**Authors:** Chao Liu, Bin Tang, Can Gao, Jianjun Deng, Min Shen, Chaolin Li, Zekun Fu, Zhan Gao, Qi Jiang, Hao Shi, Miao He, Huaiwu Jiang, Xu Jia

**Affiliations:** ^1^Non-coding RNA and Drug Discovery Key Laboratory of Sichuan Province, Chengdu Medical College, Chengdu, China; ^2^Basic Medical School, Chengdu Medical College, Chengdu, China; ^3^The First Affiliated Hospital of Chengdu Medical College, Chengdu, China; ^4^Yan'an Key Laboratory of Microbial Drug Innovation and Transformation, School of Basic Medicine, Yan'an University, Yan'an, China; ^5^Sichuan Mianyang 404 Hospital, Mianyang, China; ^6^Jinniu Maternity and Child Health Hospital of Chengdu, Chengdu, China; ^7^Institute of Blood Transfusion, Chinese Academy of Medical Sciences, Chengdu, China

**Keywords:** SARS coronavirus, virus classification, BLAST algorithm, biostatistics and bioinformatics, clustal analysis

## Abstract

The pandemic caused by the severe acute respiratory syndrome coronavirus 2 (SARS-CoV-2) has been basically under control in China since March 2020, but the import of domestic SARS-CoV-2 has begun to increase. This study reported the first case of asymptomatic SARS-CoV-2 infection imported from Spain into Sichuan Province, China, on March 11, 2020. The infected male had a body temperature of 37.5°C, normal blood oxygen saturation levels, and a computed tomography (CT) examination showed that his lungs had no shadows. However, a throat swab from the subject tested positive for SARS-CoV-2 using qPCR assay. In this study, we conducted transcriptome sequencing on respiratory throat swabs from the subject and found that the dominant SARS-CoV-2 sequence (Gene Bank ID: MW301121) was a spike protein D614G mutant strain, which is currently popular throughout world. We downloaded and analyzed SARS-CoV-2 sequences collected from cases in China and Spain for comparison and tracing purposes. After March 11, 2020, the Chinese domestic clade was naturally divided into the imported SARS-CoV-2 D614G mutant strain and evolutionarily-related similar sequences and that of sequences collected in the original Wuhan area. The sequence reported in this study was located on a small branch, far from the evolution of Wuhan sequences. As expected, the identified sequence was closely related to the evolution of the SARS-CoV-2 D614G mutant strain circulating in Spain.

## Introduction

The International Commission on Classification of Viruses (ICTV) officially classified the new virus as severe acute respiratory syndrome coronavirus 2 (SARS-CoV-2) (1). The epidemic led the Chinese government to take drastic measures to restrain the spread, including quarantining millions of residents in Wuhan and other affected cities, as well as strict designated quarantining of returning business people and students.

The global spread of SARS-CoV-2 has caused immeasurable losses to healthy living and economic development around the world (2). The WHO declared the disease a pandemic on March 11, 2020 (3). Till November 1, 2021, the total number of confirmed SARS-CoV-2 infections worldwide has exceeded 240 million, and the cumulative death has exceeded 5 million. In addition, the number of infections and deaths continues to increase.

Fortunately, under the control of the Chinese government, the epidemic in Hubei Province was effectively controlled in mid-March 2020 (4). However, inevitably, business travelers and students infected overseas caused small-scale infections after returning home. In this study, we used next-generation sequencing analysis to examine the SARS-CoV-2 infection strain from an asymptomatic individual who had returned from Spain to Sichuan Province, China, on March 11, 2020. Unexpectedly, this sequence contained a spike protein mutation (D614G). In addition, an Nsp12 mutation (P323L) and N protein double mutations (R203K and G204R) were also found. Phylogenetic tree analysis revealed that the virus sequence had very high homology with most of the sequences of infected persons who returned to China from other countries in March. Mutant strains have appeared one after another around the world, currently mainly including D614G, B.1.1.207, 501Y.V2/B.1.351, B.1.1.7, B.1.429, P.1, and P.2, B.1.617, etc. Since discovering, these mutant strains quickly spread to dozens of countries including the United States, Singapore, and the United Kingdom, and tend to become the main epidemic strains in some areas of these countries. To trace the source of such RNA virus infection, genomics testing is of great significance to epidemiological investigation and monitoring. In addition, the presence of fewer nucleotide (nt) mutations in the viral genome of asymptomatic patients in cluster infection may help us clarify the virus shedding pattern and replication model of SARS-CoV-2 infection.

## Materials and Methods

### SARS-CoV-2 RNA Extraction and Real-Time Quantitative PCR

The samples tested for SARS-CoV-2 included oropharyngeal swabs, sputum, and stool. RNA was extracted in a biosafety II laboratory using a TRIzol LS kit (Thermo Fisher Scientific, Waltham, MA, USA), according to the manufacturer's instructions. According to the guidelines released by the National Health Commission of the People's Republic of China (NHC of China), COVID-19 (SARS-CoV-2) Nucleic Acid Testing Kit (Bioer Technology, Hangzhou, Zhejiang, China) was used to detect SARS-CoV-2 genes, including the ORF1ab polyprotein gene (ORF1ab) and the nucleoprotein (N) gene. ORF1ab Fluorescent probe (P): 5′-FAM-CCGTCTGCGGTATGTGGAAAGGTTATGG-BHQ1-3′, N Fluorescent probe (P): 5′-FAM-TTGCTGCTGCTTGACAGATT-TAMRA-3′.

### SARS-CoV-2 Gene Sequencing

The samples with SARS-CoV-2 positive were analyzed by quantitative reverse transcription PCR (RT-qPCR) from the subject's respiratory secretions and stools, and the next-generation sequencing was used for sequencing. The sequencing library was constructed using a transposase-based methodology and an Illumina Hiseq 4500 sequencing platform (Illumina, San Diego, CA, USA). At least 20 million paired-end 150-bp reads were generated for each sample.

Quality control processes included adapter trimming, low-quality read removal, and short read removal by fastp v0.20.0 (5). Processed reads from each sample were first aligned to the human hg38 genome (GCA_000001405.20) using hisat2 v2.1.0 (6). Only unassigned reads were exported to a bam file using samtools v1.9 (-f 4 parameter). Non-human reads were converted back to FASTQ format and mapped to SARS-CoV-2 (GenBank: MN908947.37) using BWA mem v0.7.17.8. Only mapped reads were extracted using samtools. Duplicate reads were removed using Picard Toolkit v2.22.1.9. Binary BCF files were generated using samtools mpileup v1.9 and then intra-individual variants were called using VarScan v2.3.9.10. Variants in each sample were filtered using bcftools v1.10.2 based on satisfying the following criteria: (i) read depth at a particular position ≥20, (ii) conditional genotype quality (GQ) ≥20, and (iii) no other sites of variation in the adjacent five bases. Genetic variant annotation and functional effect prediction were carried out using SnpEff v4.3t11 with the GFF file of reference genome.

### Phylogenetic Analysis

All of the available SARS-CoV-2 genome sequences (screening conditions were “complete” and “high coverage”) were downloaded from the GISAID database (https://www.epicov.org/epi3/frontend). MEGA software v7.018 was used to perform multiple sequence alignment between the sequence sequenced by the subject and the available GISAID sequence.

Phylogenetic trees were generated using MEGA software 18 with the neighbor joining method and Kimura-2-parameter as the nt substitution model, and performing 1,000 bootstrap replications (7).

## Results

### Overview of Case Information

**Figure 1:**
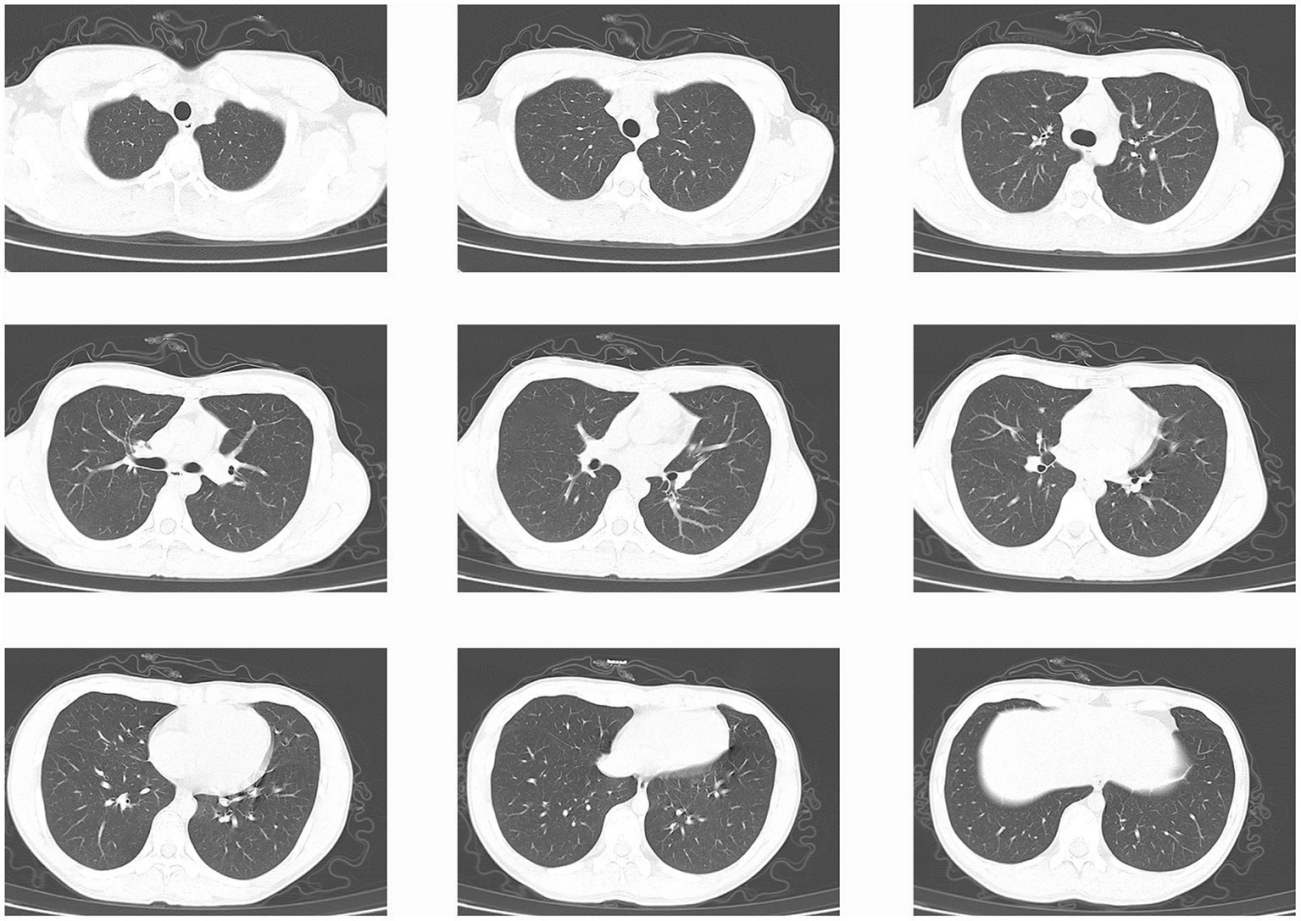
Chest computed tomography **(CT)** scan of the positive SARS-CoV-2 individual examined on March 13, 2020. The scans show healthy lungs based on the absence of abnormal shadow patch and the presence of ground glass shadow.

On March 11, 2020, a male citizen who had lived in Spain for more than 6 months, returned from Barcelona to Sichuan, China. According to the request of Chinese government, the individual was quarantined after returning to China and throat swab was obtained for SARS-CoV-2 screening. On March 13, RT-qPCR amplification of both the SARS-CoV-2 ORF1ab gene and N gene revealed positive results. However, the subject did not display any discomfort and his body temperature was 37.5°C. Soon, he was transferred to a designated hospital for isolation and treatment. Later blood test performed in the hospital showed a decrease in lymphocytes (0.73 ^*^ 10^9^/L) and an increase in monocytes (0.75 ^*^ 10^9^/L). However, the patient's blood oxygen saturation level was normal, the lung computed tomography (CT) scan was normal (Figure 1), and the presence of other common pathogens/infectious viruses was ruled out.

A treatment plan was designed for the infected patient after referring to the seventh edition of “Diagnosis and Treatment Plan for Pneumonia Caused by New Coronavirus Infection.” The treatment consisted of interferon α-2β nebulized inhalation, lopinavir/ritonavir, and “LianHua Qingwen granules” for antiviral therapy and other symptomatic treatments. On March 30, RT-qPCR testing for the SARS-CoV-2 ORF1ab gene and N gene yielded negative results. On April 2, a serum SARS-CoV-2 antibody test was positive. After 14 days of consecutive testing of the patient's feces and throat swabs, RT-qPCR results were negative for the SARS-CoV-2 ORF1ab gene and N gene, and the patient was discharged.

### Whole Genome Sequencing Analysis

To analyze the characteristics of the SARS-CoV-2 sequence, we performed next-generation sequencing on the collected respiratory and fecal samples. The complete sequencing data for the SARS-CoV-2 strain from the infected subject has been submitted to NCBI (Gene Bank ID: MW301121). The sequence contained a total of 29,903 bases. The sequence of the SARS-CoV-2 strain was compared with the hCoV-19/Wuhan/WIV04/2019 (GISAID Accession ID: EPI_ISL_479681) for mutation analysis. Eight single nucleotide polymorphisms (SNPs) were identified, including C242T, C313T, C3037T, C14408T, A23403G, G28881A, G28882A, and G28883C. In addition, these mutations were expressed as amino acid changes in the spike (D614G), Nsp12 (P323L), and N proteins (R203K and G204R). According to the evolutionary classification of the GISAID database, the new sequence identified in this study belonged to the B.1.1 lineage.

### Phylogenetic Analysis

**Figure 2:**
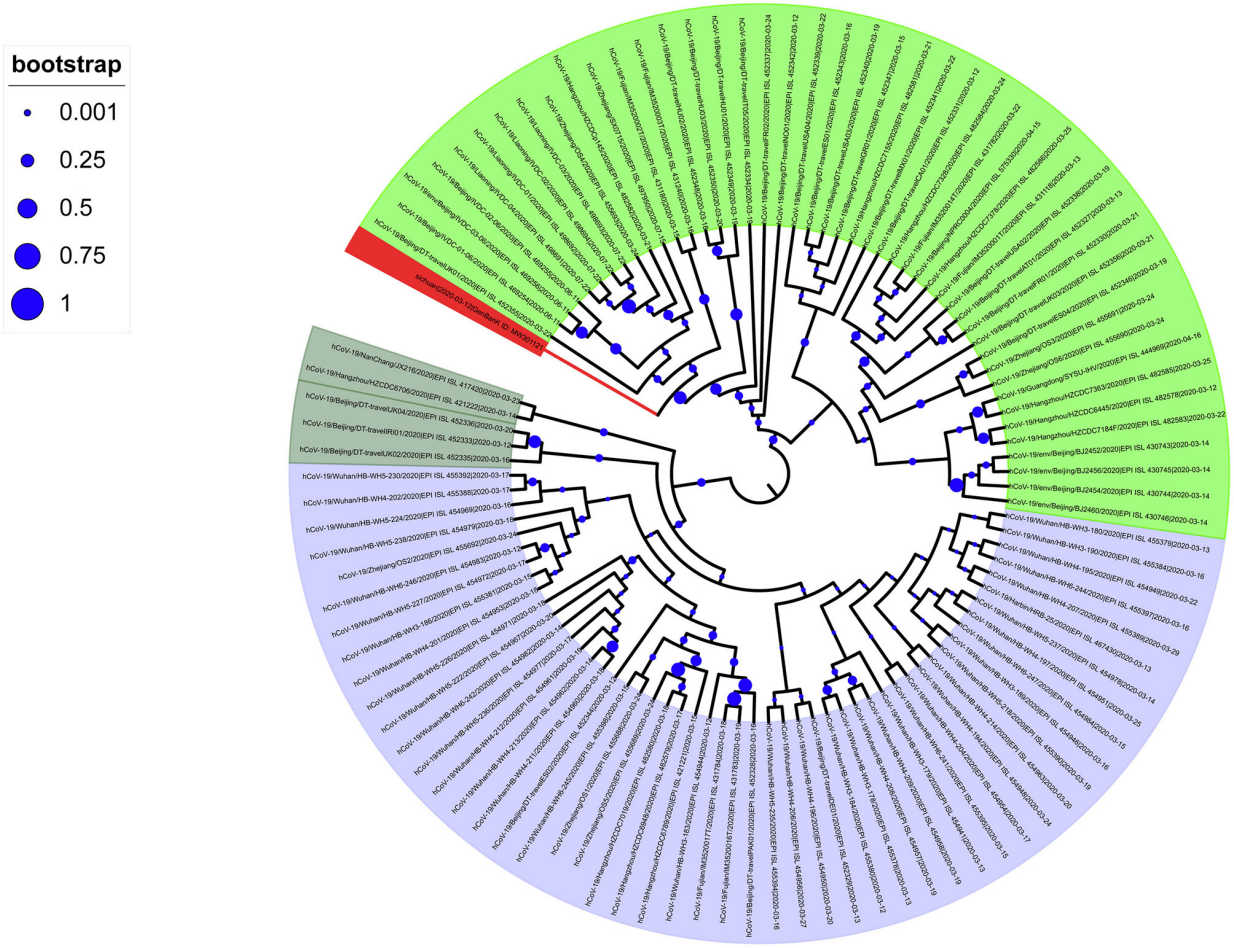
Phylogenetic tree of SARS-CoV-2 sequences collected in China after March 11, 2020. The SARS-CoV-2 of the Wuhan series is Branch A (light blue); the next-generation sequencing sequence (Gene Bank ID: MW301121) was stained red.

To analyze the imported SARS-CoV-2 cases, we downloaded all high-quality and complete SARS-CoV-2 sequences uploaded in China after March 12 from the GISAID database. The evolutionary tree showed that the phylogenetic tree had two large clades: branch A, contained a concentration of Wuhan sequences (shaded light blue in Figure 2); and input branch B, grouped sequences from multiple regions (Figure 2). In addition, there were scattered evolutionary branches from oversea residents who had returned to China. The evolutionary tree showed that the domestic SARS-CoV-2 epidemic was relatively conserved, but the imported SARS-CoV-2 sequences showed diversity. Among the latter, SARS-CoV-2 with the D614G spike protein mutation was the main input.

**Figure 3:**
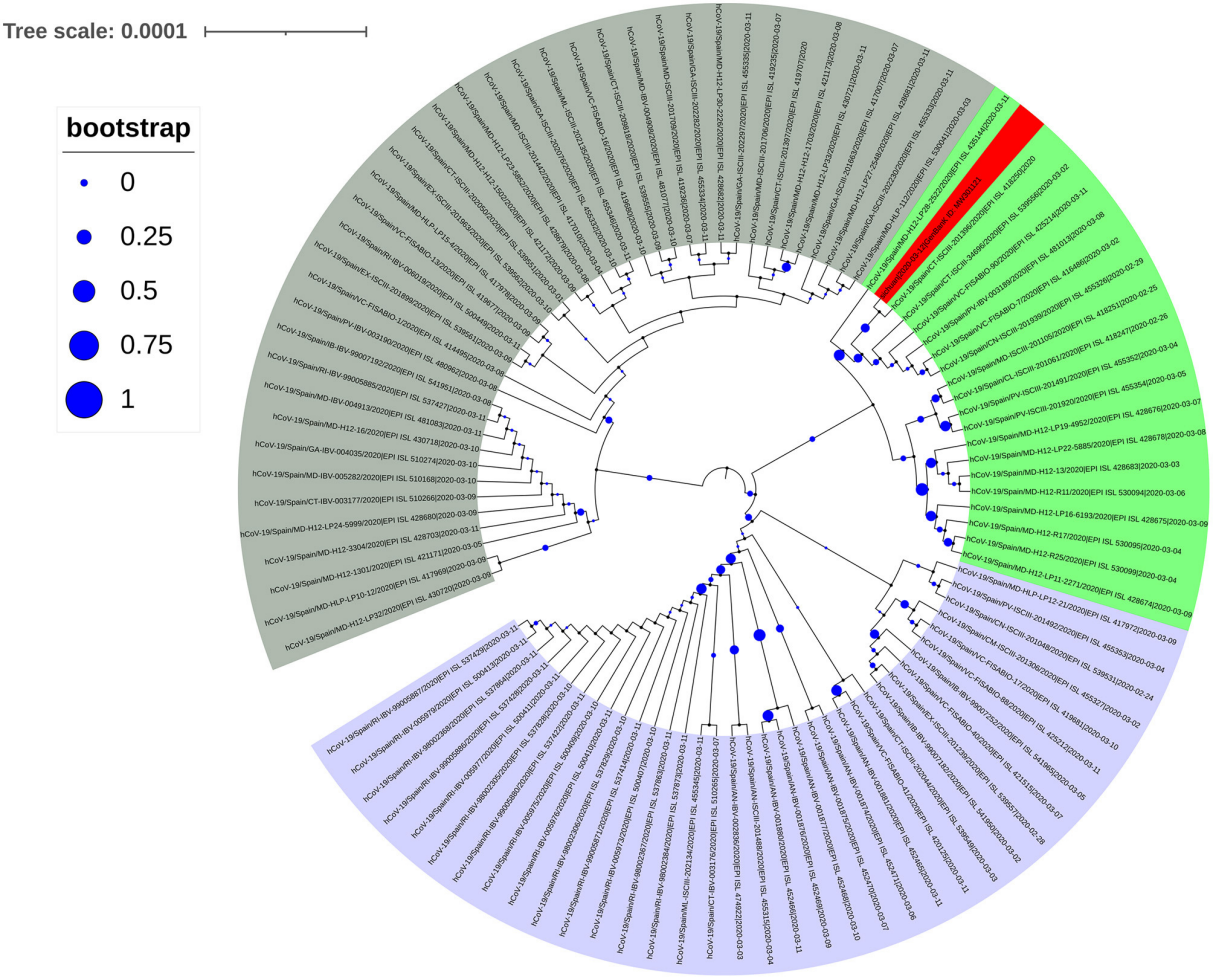
Phylogenetic tree of the SARS-CoV-2 spike protein sequence with the D614G mutation collected in Spain before March 11, 2020. The next-generation sequencing sequence (Gene Bank ID: MW301121) was stained red.

The spike protein in SARS-CoV-2 interacts with the human ACE2 receptor to gain entry into a cell to initiate infection. Previous studies found that SARS-CoV-2 sequences with the D614G mutation were predominant in European countries (8). To analyze the source of SARS-CoV-2 of the infected subject from Spain, we first performed a simple analysis against sequences uploaded to the GISAID database in Spain before March 11. A total of 545 sequences (including incomplete and low-quality sequences) were uploaded, of which 108 sequences had D614G mutation in the spike protein, and 96 sequences had P323L mutation in NSP12. We downloaded the complete high-quality SARS-CoV-2 sequences of all spike protein D614G mutations in Spain before March 11, and used them to construct a phylogenetic tree (Figure 3). Analysis of this tree did not reveal any obvious dominant branches in Spain. The mixed D614G mutation and other multiple mutations formed multiple branches. Some of these branches formed small-scale clusters, including the new Sichuan sequence (shaded red in Figure 3) that we isolated and sequenced. However, this branch did not form a larger dominant epidemic group.

## Discussion

Severe acute respiratory syndrome coronavirus 2 is by far the most serious pandemic of this century. Severe acute respiratory syndrome coronavirus 2 was first reported in Wuhan, China, in December 2019 (9). However, due to the efforts of the Chinese government and their health system, the large-scale epidemic in China basically ended by late March 2020 (10). Since then, all business travelers and students returned to China, were required to be quarantined and COVID-19 patients were treated within the country. As a result, the virus from these returning carriers has not caused a new large-scale epidemic in China. In fact, only 118 SARS-CoV-2 sequences were uploaded to the GISAID database in China since late March 2020.

In this study, we sequenced the SARS-CoV-2 virus carried by a Chinese citizen who lived in Spain and returned to China in March 2020. A total of eight bases in the viral RNA sequence were mutated (referred to as: Gene Bank ID: MW301121) as follows: C241T, C313T, C3037T, C14408T, A23403G, and GGG28881AAC. These changes translated to four amino acid changes in three proteins: spike (D614G), Nsp12 (P323L), and N protein (R203K and G204R). Analysis determined that the modified sequence belonged to the B.1.1 lineage, and only 20 sequences of this lineage in China have been sequenced and uploaded, indicating that the SARS-CoV-2 strain in the B.1.1 lineage is not the main epidemic viral strain in China. Previous reports indicated that the B.1.1 lineage is one of the most dominant lineages in continental Europe and the United States (11). Although it has been found that SARS-CoV-2 with the D614G mutation in the spike protein can replicate and spread faster than strains without this mutation (12), interestingly, the P323L mutation has been reported to co-evolve with D614G worldwide, this adaptation of the virus might strengthen SARS-CoV-2 G614 strain replication rates and infectivity (12). Siqi suggested that R203K and G204R mutations could change viral protein structure, binding affinity, and hot spots of the interface, thereby impact on SARS-CoV-2 transmission, diagnosis, and treatment of COVID-19 (13). The strict isolation, observation, and treatment were very effective in controlling the spread of SARS-CoV-2 from person to person, regardless of the strain.

Most of the SARS-CoV-2 sequences collected after March 12, 2020, in China belong to the B lineage, of which 44 sequences belong to the B.1 lineage. Further analysis found that these B.1 genealogies mainly sampled from patients in Beijing, Zhejiang, Fujian, and Liaoning. Among them, the small clusters of infectious cases in Beijing (14) and Liaoning have closely contacted to imported frozen food contaminated with SARS-CoV-2. In addition, in our evolutionary tree, our new sequence is close to the sequences of Beijing and Liaoning, much closer than the sequences of Wuhan. This study showed that from March 12 onwards, the infection caused by SARS-CoV-2 in China could be divided into two parts: the local clade based on Wuhan native sequences and the main clade formed by the D614G mutation and many other different clades. Thus, the SARS-CoV-2 strain introduced to China after March 12 is not a single introduction, and its genetic background is relatively complicated.

We determined the Sichuan-2020 sequence belongs to haplogroup A2a4 (diagnostic variants: C241T–C3037T–C14408T–A23403G plus characteristic MNP: GGG28881AAC) according to the haplogroup nomenclature of SARS-CoV-2 clades by Gómez-Carballa et al. (15) Haplogroup A2a4 is one of the most successful clades worldwide, and A2a4 genomes have been identified in 63 different countries, areas, and territories (16). In the Spanish evolutionary tree, the A2a4 haplogroup did not form a dominant spread in Spain (Figure 2). According to previous reports, the A2a4 haplogroup has the highest frequency in Europe and Russia, and its earliest genome data appeared in European countries for the first time on February 24, 2020 (16). It accumulated in Spain mainly from March 11 to March 28, 2020 (59 out of 68 A2a4 genomes) (16). This basically coincides with the time the carrier in our study returned to China, indicating that the SARS-CoV-2 strain introduced to China was originated from Spain, although Spain may not be the first country where the D614G mutation was discovered.

In summary, we sequenced and reported the first case of mutations in the genes encoding the spike (D614G) and Nsp12 (P323L) proteins of SARS-CoV-2 in Sichuan Province, China. The SARS-CoV-2 strain was sequenced from a respiratory sample from an asymptomatic Chinese citizen returning from Spain. Timely sharing of the entire genome sequence of SARS-CoV-2 strains and information on their geographic distribution over time is very important for monitoring viral genetic changes related to the spread of the virus and its clinical manifestations.

## Data Availability Statement

The datasets presented in this study can be found in online repositories. The names of the repository/repositories and accession number(s) can be found below: Gene Bank ID: MW301121.

## Ethics Statement

The studies involving human participants were reviewed and approved by Sichuan Mianyang 404 Hospital (No. 20201103). This research report complies with CARE guidelines (17). The patients/participants provided their written informed consent to participate in this study.

## Author Contributions

CLiu and BT collected the literature and wrote the article. CG revised and submitted the manuscript. JD, MS, CLi, ZF, ZG, QJ, and HS revised the article. MH, HJ, and XJ designed the study and revised the article. All authors have read and approved the final article. All authors contributed toward data analysis, drafting and critically revising the paper and agree to be accountable for all aspects of the work.

## Funding

This work was supported by grants from the National Natural Science Foundation of China (Nos. 31870135, 31600116), the 1000 Talent Plan of Sichuan Province (No. 980), 2020 Major Medical Innovation Project of Sichuan Provincial Health Commission (No. 20ZDCX002), and the Research Fund of Non-coding RNA and Drug Discovery Key Laboratory of Sichuan Province (No. FB20-03).

## Conflict of Interest

The authors declare that the research was conducted in the absence of any commercial or financial relationships that could be construed as a potential conflict of interest.

## Publisher's Note

All claims expressed in this article are solely those of the authors and do not necessarily represent those of their affiliated organizations, or those of the publisher, the editors and the reviewers. Any product that may be evaluated in this article, or claim that may be made by its manufacturer, is not guaranteed or endorsed by the publisher.
